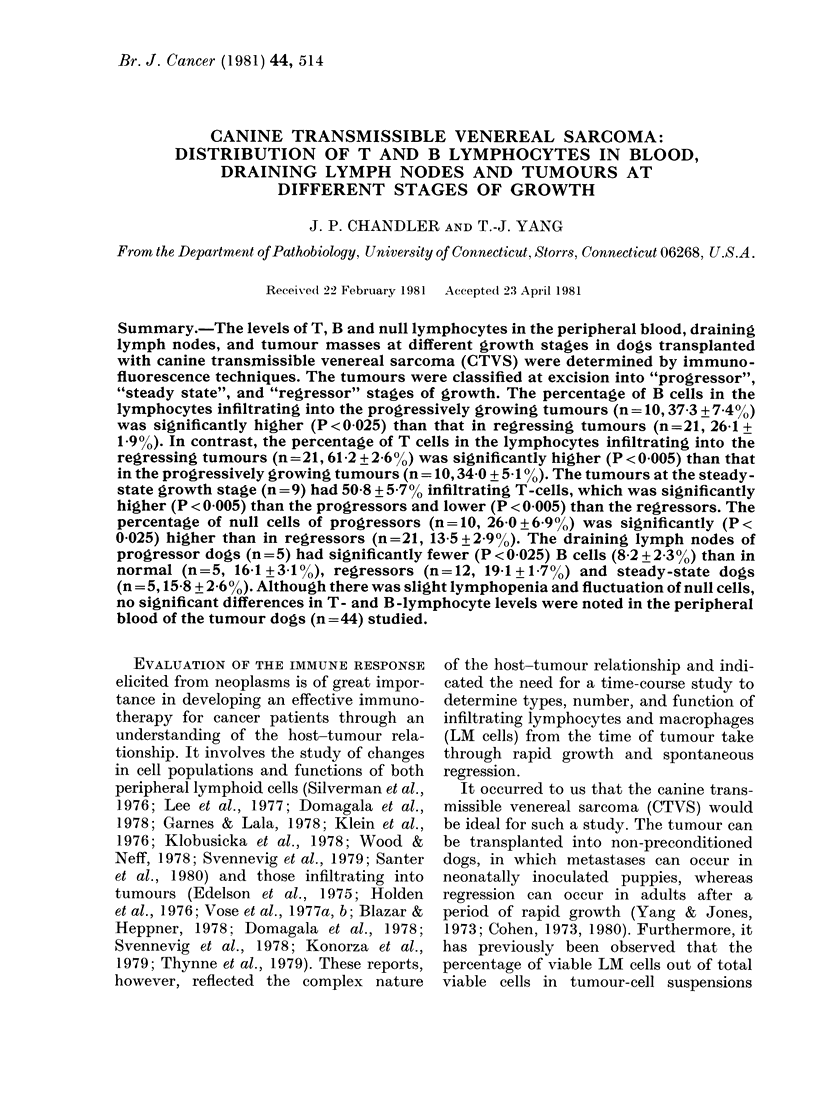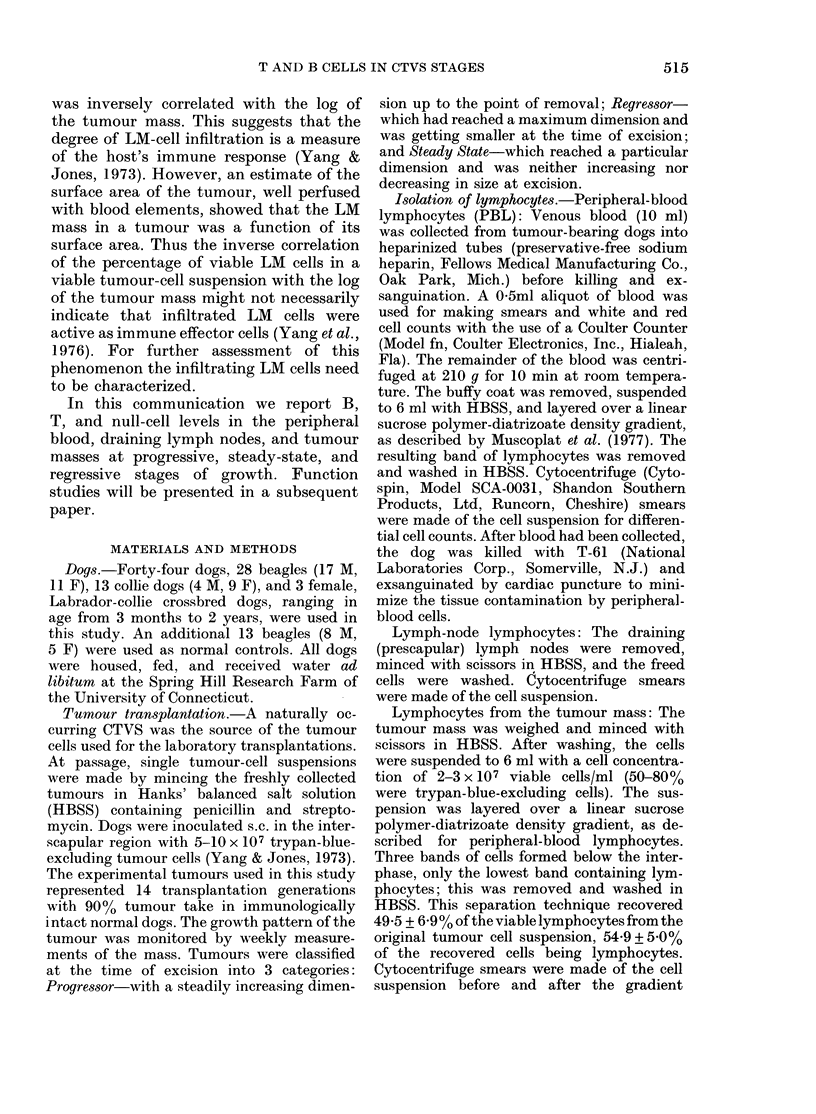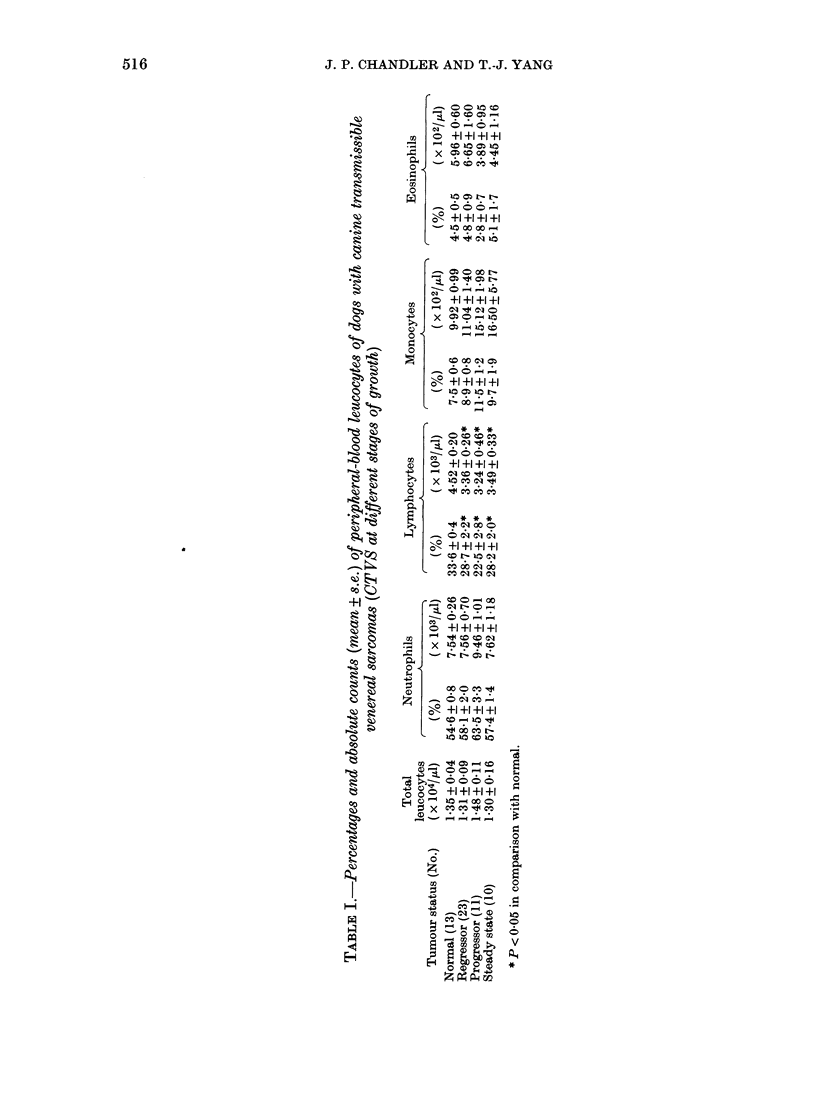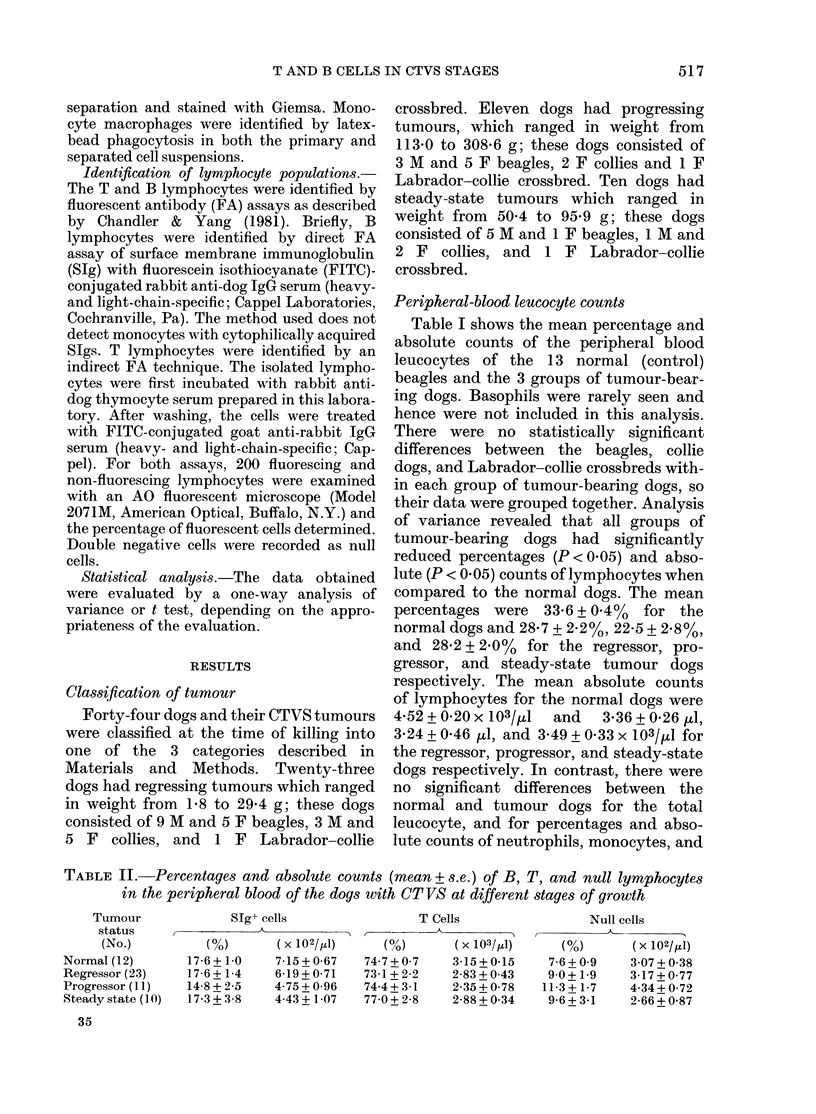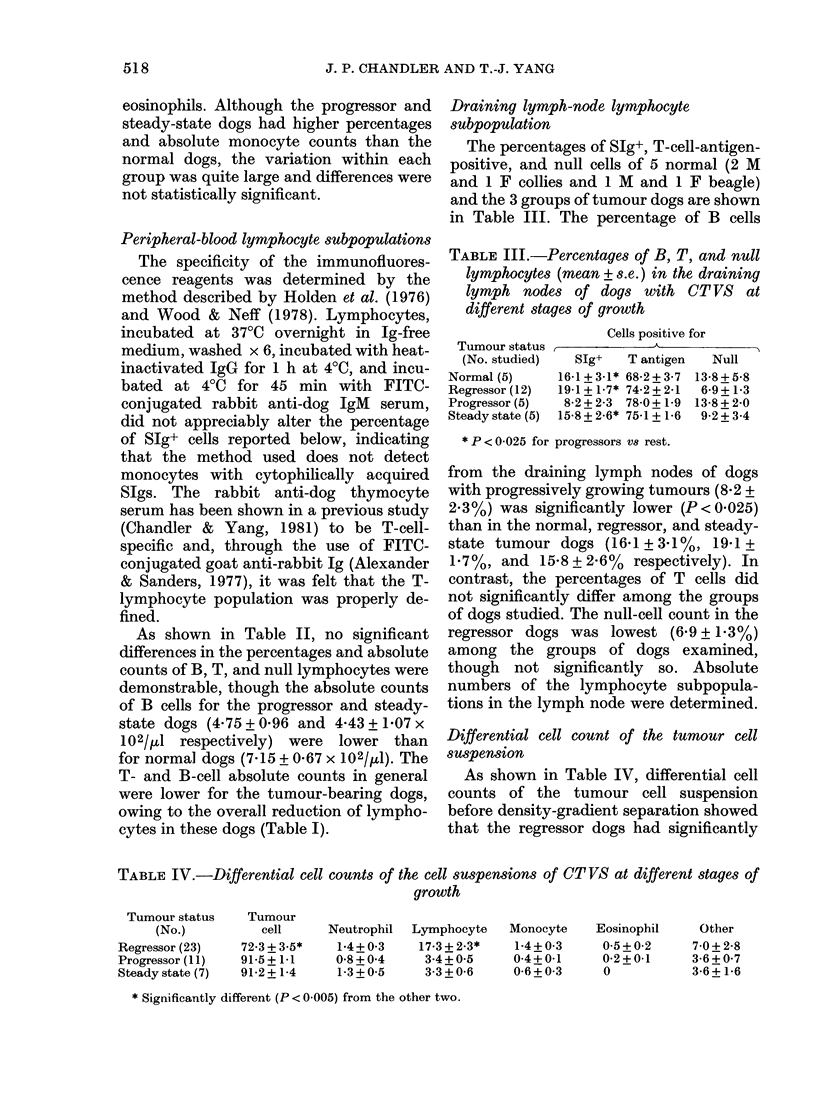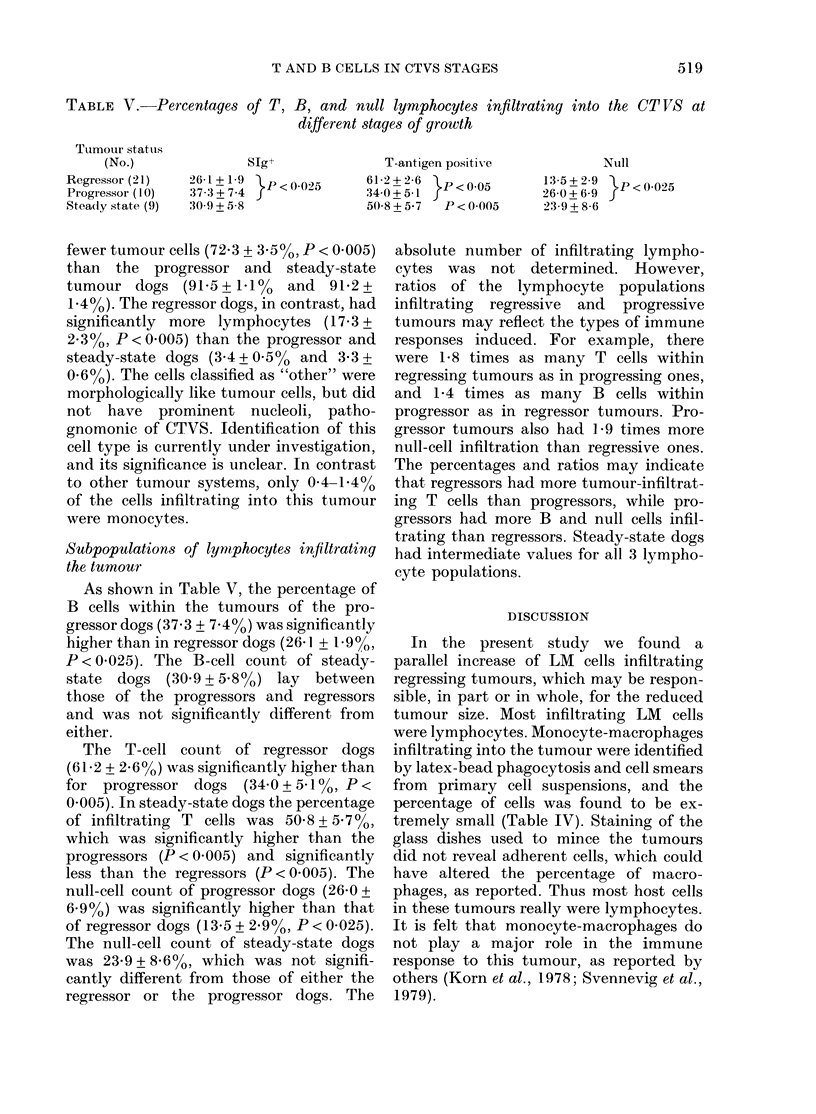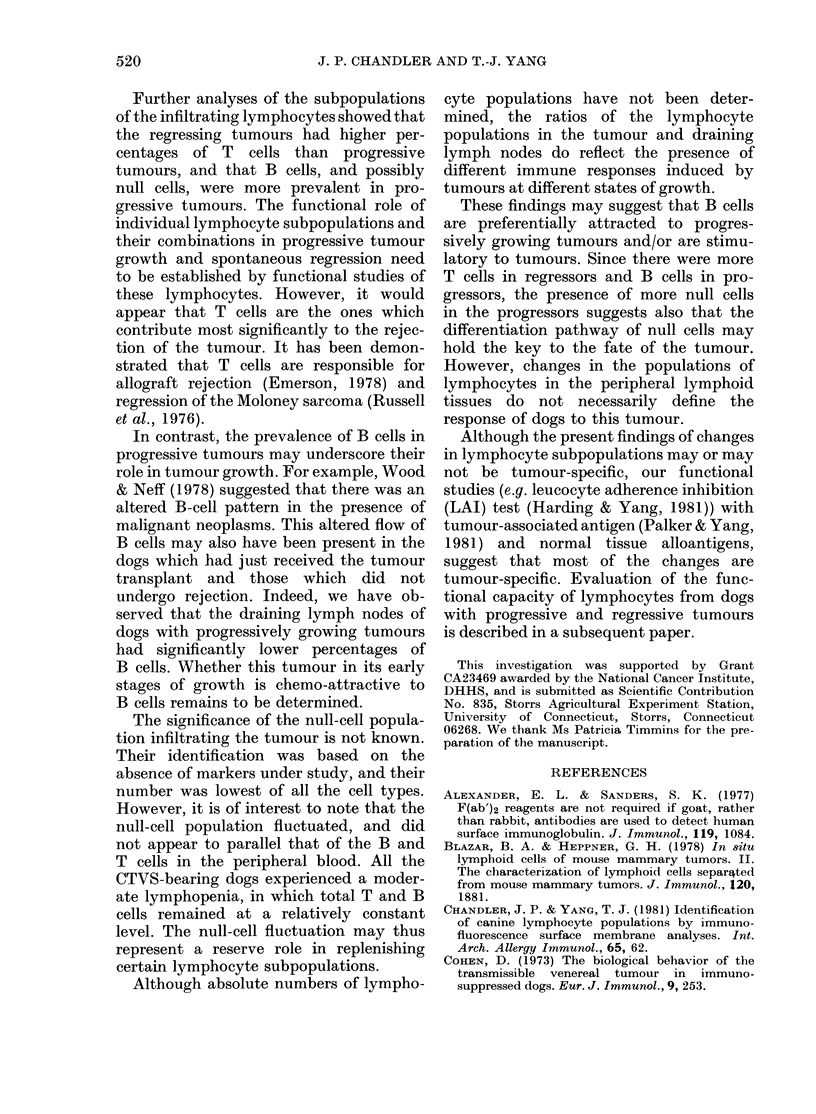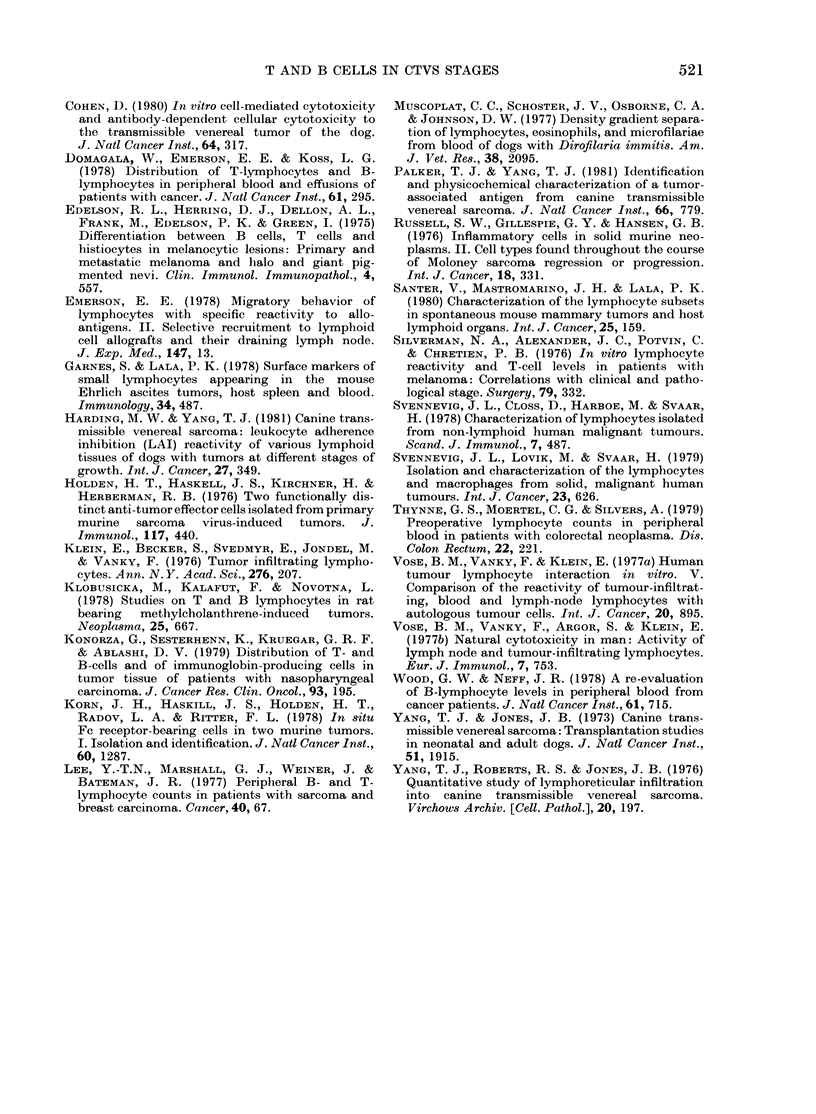# Canine transmissible venereal sarcoma: distribution of T and B lymphocytes in blood, draining lymph nodes and tumours at different stages of growth.

**DOI:** 10.1038/bjc.1981.220

**Published:** 1981-10

**Authors:** J. P. Chandler, T. J. Yang

## Abstract

The levels of T, B and null lymphocytes in the peripheral blood, draining lymph nodes, and tumour masses at different growth stages in dogs transplanted with canine transmissible venereal sarcoma (CTVS) were determined by immunofluorescence techniques. The tumours were classified at excision into "progressor", "steady state", and "regressor" stages of growth. The percentage of B cells in the lymphocytes infiltrating into the progressively growing tumours (n = 10, 37.3 +/- 7.4%) was significantly higher (P less than 0.025) than that in regressing tumours (n = 21, 26.1 +/- 1.9%). In contrast, the percentage of T cells in the lymphocytes infiltrating into the regressing tumours (n = 21, 61.2 +/- 2.6%) was significantly higher (P less than 0.005) than that in the progressively growing tumours (n = 10, 34.0 +/- 5.1%). The tumours at the steady-state growth stage (n = 9) had 50.8 +/- 5.7% infiltrating T-cells, which was significantly higher (P less than 0.005) than the progressors and lower (P less than 0.005) than the regressors. The percentage of null cells of progressors (n = 10, 26.0 +/- 6.9%) was significantly (P less than 0.025) higher than in regressors (n = 21, 13.5 +/- 2.9%). The draining lymph nodes of progressor dogs (n = 5) had significantly fewer (P less than 0.025) B cells (8.2 +/- 2.3%) than in normal (n = 5, 16.1 +/- 3.1%), regressors (n = 12, 19.1 +/- 1.7%) and steady-state dogs (n = 5, 15.8 +/- 2.6%). Although there was slight lymphopenia and fluctuation of null cells, no significant differences in T- and B-lymphocyte levels were noted in the peripheral blood of the tumour dogs (n = 44) studied.


*Br. J. Cancer* (1981) **44**, 514

### CANINE TRANSMISSIBLE VENEREAL SARCOMA: DISTRIBUTION OF T AND B LYMPHOCYTES IN BLOOD, DRAINING LYMPH NODES AND TUMOURS AT DIFFERENT STAGES OF GROWTH

J. P. CHANDLER AND T.-J. YANG

*From the Department of Pathobiology, University of Connecticut, Storrs, Connecticut* 06268, *U.S.A.*

Received 22 February 1981 Accepted 23 April 1981

**Summary.—The levels of T, B and null lymphocytes in the peripheral blood, draining lymph nodes, and tumour masses at different growth stages in dogs transplanted with canine transmissible venereal sarcoma (CTVS) were determined by immunofluorescence techniques. The tumours were classified at excision into "progressor", "steady state", and "regressor" stages of growth. The percentage of B cells in the lymphocytes infiltrating into the progressively growing tumours ($n = 10, 37{\cdot}3 \pm 7{\cdot}4\%$) was significantly higher ($P < 0{\cdot}025$) than that in regressing tumours ($n = 21, 26{\cdot}1 \pm 1{\cdot}9\%$). In contrast, the percentage of T cells in the lymphocytes infiltrating into the regressing tumours ($n = 21, 61{\cdot}2 \pm 2{\cdot}6\%$) was significantly higher ($P < 0{\cdot}005$) than that in the progressively growing tumours ($n = 10, 34{\cdot}0 \pm 5{\cdot}1\%$). The tumours at the steady-state growth stage ($n = 9$) had $50{\cdot}8 \pm 5{\cdot}7\%$ infiltrating T-cells, which was significantly higher ($P < 0{\cdot}005$) than the progressors and lower ($P < 0{\cdot}005$) than the regressors. The percentage of null cells of progressors ($n = 10$, $26{\cdot}0 \pm 6{\cdot}9\%$) was significantly ($P < 0{\cdot}025$) higher than in regressors ($n = 21$, $13{\cdot}5 \pm 2{\cdot}9\%$). The draining lymph nodes of progressor dogs ($n = 5$) had significantly fewer ($P < 0{\cdot}025$) B cells ($8{\cdot}2 \pm 2{\cdot}3\%$) than in normal ($n = 5$, $16{\cdot}1 \pm 3{\cdot}1\%$), regressors ($n = 12$, $19{\cdot}1 \pm 1{\cdot}7\%$) and steady-state dogs ($n = 5, 15{\cdot}8 \pm 2{\cdot}6\%$). Although there was slight lymphopenia and fluctuation of null cells, no significant differences in T- and B-lymphocyte levels were noted in the peripheral blood of the tumour dogs ($n = 44$) studied.**

Evaluation of the immune response elicited from neoplasms is of great importance in developing an effective immunotherapy for cancer patients through an understanding of the host–tumour relationship. It involves the study of changes in cell populations and functions of both peripheral lymphoid cells (Silverman *et al.*, 1976; Lee *et al.*, 1977; Domagala *et al.*, 1978; Garnes & Lala, 1978; Klein *et al.*, 1976; Klobusicka *et al.*, 1978; Wood & Neff, 1978; Svennevig *et al.*, 1979; Santer *et al.*, 1980) and those infiltrating into tumours (Edelson *et al.*, 1975; Holden *et al.*, 1976; Vose *et al.*, 1977*a*, *b*; Blazar & Heppner, 1978; Domagala *et al.*, 1978; Svennevig *et al.*, 1978; Konorza *et al.*, 1979; Thynne *et al.*, 1979). These reports, however, reflected the complex nature of the host–tumour relationship and indicated the need for a time-course study to determine types, number, and function of infiltrating lymphocytes and macrophages (LM cells) from the time of tumour take through rapid growth and spontaneous regression.

It occurred to us that the canine transmissible venereal sarcoma (CTVS) would be ideal for such a study. The tumour can be transplanted into non-preconditioned dogs, in which metastases can occur in neonatally inoculated puppies, whereas regression can occur in adults after a period of rapid growth (Yang & Jones, 1973; Cohen, 1973, 1980). Furthermore, it has previously been observed that the percentage of viable LM cells out of total viable cells in tumour-cell suspensions was inversely correlated with the log of the tumour mass. This suggests that the degree of LM-cell infiltration is a measure of the host's immune response (Yang & Jones, 1973). However, an estimate of the surface area of the tumour, well perfused with blood elements, showed that the LM mass in a tumour was a function of its surface area. Thus the inverse correlation of the percentage of viable LM cells in a viable tumour-cell suspension with the log of the tumour mass might not necessarily indicate that infiltrated LM cells were active as immune effector cells (Yang *et al.*, 1976). For further assessment of this phenomenon the infiltrating LM cells need to be characterized.

In this communication we report B, T, and null-cell levels in the peripheral blood, draining lymph nodes, and tumour masses at progressive, steady-state, and regressive stages of growth. Function studies will be presented in a subsequent paper.

### MATERIALS AND METHODS

*Dogs*.—Forty-four dogs, 28 beagles (17 M, 11 F), 13 collie dogs (4 M, 9 F), and 3 female, Labrador-collie crossbred dogs, ranging in age from 3 months to 2 years, were used in this study. An additional 13 beagles (8 M, 5 F) were used as normal controls. All dogs were housed, fed, and received water *ad libitum* at the Spring Hill Research Farm of the University of Connecticut.

*Tumour transplantation*.—A naturally occurring CTVS was the source of the tumour cells used for the laboratory transplantations. At passage, single tumour-cell suspensions were made by mincing the freshly collected tumours in Hanks' balanced salt solution (HBSS) containing penicillin and streptomycin. Dogs were inoculated s.c. in the interscapular region with $5–10 \times 10^7$ trypan-blue-excluding tumour cells (Yang & Jones, 1973). The experimental tumours used in this study represented 14 transplantation generations with 90% tumour take in immunologically intact normal dogs. The growth pattern of the tumour was monitored by weekly measurements of the mass. Tumours were classified at the time of excision into 3 categories: *Progressor*—with a steadily increasing dimension up to the point of removal; *Regressor*—which had reached a maximum dimension and was getting smaller at the time of excision; and *Steady State*—which reached a particular dimension and was neither increasing nor decreasing in size at excision.

*Isolation of lymphocytes*.—Peripheral-blood lymphocytes (PBL): Venous blood (10 ml) was collected from tumour-bearing dogs into heparinized tubes (preservative-free sodium heparin, Fellows Medical Manufacturing Co., Oak Park, Mich.) before killing and exsanguination. A 0·5ml aliquot of blood was used for making smears and white and red cell counts with the use of a Coulter Counter (Model fn, Coulter Electronics, Inc., Hialeah, Fla). The remainder of the blood was centrifuged at 210 *g* for 10 min at room temperature. The buffy coat was removed, suspended to 6 ml with HBSS, and layered over a linear sucrose polymer-diatrizoate density gradient, as described by Muscoplat *et al.* (1977). The resulting band of lymphocytes was removed and washed in HBSS. Cytocentrifuge (Cytospin, Model SCA-0031, Shandon Southern Products, Ltd, Runcorn, Cheshire) smears were made of the cell suspension for differential cell counts. After blood had been collected, the dog was killed with T-61 (National Laboratories Corp., Somerville, N.J.) and exsanguinated by cardiac puncture to minimize the tissue contamination by peripheral-blood cells.

Lymph-node lymphocytes: The draining (prescapular) lymph nodes were removed, minced with scissors in HBSS, and the freed cells were washed. Cytocentrifuge smears were made of the cell suspension.

Lymphocytes from the tumour mass: The tumour mass was weighed and minced with scissors in HBSS. After washing, the cells were suspended to 6 ml with a cell concentration of $2–3 \times 10^7$ viable cells/ml (50–80% were trypan-blue-excluding cells). The suspension was layered over a linear sucrose polymer-diatrizoate density gradient, as described for peripheral-blood lymphocytes. Three bands of cells formed below the interphase, only the lowest band containing lymphocytes; this was removed and washed in HBSS. This separation technique recovered $49·5 \pm 6·9$% of the viable lymphocytes from the original tumour cell suspension, $54·9 \pm 5·0$% of the recovered cells being lymphocytes. Cytocentrifuge smears were made of the cell suspension before and after the gradient

TABLE I.—*Percentages and absolute counts (mean ± s.e.) of peripheral-blood leucocytes of dogs with canine transmissible venereal sarcomas (CTVS at different stages of growth)*

| Tumour status (No.) | Total leucocytes ($\times 10^4/\mu l$) | Neutrophils (%) | Neutrophils ($\times 10^3/\mu l$) | Lymphocytes (%) | Lymphocytes ($\times 10^3/\mu l$) | Monocytes (%) | Monocytes ($\times 10^2/\mu l$) | Eosinophils (%) | Eosinophils ($\times 10^2/\mu l$) |
|---|---|---|---|---|---|---|---|---|---|
| Normal (13) | 1·35 ± 0·04 | 54·6 ± 0·8 | 7·54 ± 0·26 | 33·6 ± 0·4 | 4·52 ± 0·20 | 7·5 ± 0·6 | 9·92 ± 0·99 | 4·5 ± 0·5 | 5·96 ± 0·60 |
| Regressor (23) | 1·31 ± 0·09 | 58·1 ± 2·0 | 7·56 ± 0·70 | 28·7 ± 2·2* | 3·36 ± 0·26* | 8·9 ± 0·8 | 11·04 ± 1·40 | 4·8 ± 0·9 | 6·65 ± 1·60 |
| Progressor (11) | 1·48 ± 0·11 | 63·5 ± 3·3 | 9·46 ± 1·01 | 22·5 ± 2·8* | 3·24 ± 0·46* | 11·5 ± 1·2 | 15·12 ± 1·98 | 2·8 ± 0·7 | 3·89 ± 0·95 |
| Steady state (10) | 1·30 ± 0·16 | 57·4 ± 1·4 | 7·62 ± 1·18 | 28·2 ± 2·0* | 3·49 ± 0·33* | 9·7 ± 1·9 | 16·50 ± 5·77 | 5·1 ± 1·7 | 4·45 ± 1·16 |

* $P < 0·05$ in comparison with normal.

separation and stained with Giemsa. Monocyte macrophages were identified by latex-bead phagocytosis in both the primary and separated cell suspensions.

*Identification of lymphocyte populations.*—The T and B lymphocytes were identified by fluorescent antibody (FA) assays as described by Chandler & Yang (1981). Briefly, B lymphocytes were identified by direct FA assay of surface membrane immunoglobulin (SIg) with fluorescein isothiocyanate (FITC)-conjugated rabbit anti-dog IgG serum (heavy- and light-chain-specific; Cappel Laboratories, Cochranville, Pa). The method used does not detect monocytes with cytophilically acquired SIgs. T lymphocytes were identified by an indirect FA technique. The isolated lymphocytes were first incubated with rabbit anti-dog thymocyte serum prepared in this laboratory. After washing, the cells were treated with FITC-conjugated goat anti-rabbit IgG serum (heavy- and light-chain-specific; Cappel). For both assays, 200 fluorescing and non-fluorescing lymphocytes were examined with an AO fluorescent microscope (Model 2071M, American Optical, Buffalo, N.Y.) and the percentage of fluorescent cells determined. Double negative cells were recorded as null cells.

*Statistical analysis.*—The data obtained were evaluated by a one-way analysis of variance or *t* test, depending on the appropriateness of the evaluation.

### RESULTS

### *Classification of tumour*

Forty-four dogs and their CTVS tumours were classified at the time of killing into one of the 3 categories described in Materials and Methods. Twenty-three dogs had regressing tumours which ranged in weight from 1·8 to 29·4 g; these dogs consisted of 9 M and 5 F beagles, 3 M and 5 F collies, and 1 F Labrador–collie crossbred. Eleven dogs had progressing tumours, which ranged in weight from 113·0 to 308·6 g; these dogs consisted of 3 M and 5 F beagles, 2 F collies and 1 F Labrador–collie crossbred. Ten dogs had steady-state tumours which ranged in weight from 50·4 to 95·9 g; these dogs consisted of 5 M and 1 F beagles, 1 M and 2 F collies, and 1 F Labrador–collie crossbred.

### *Peripheral-blood leucocyte counts*

Table I shows the mean percentage and absolute counts of the peripheral blood leucocytes of the 13 normal (control) beagles and the 3 groups of tumour-bearing dogs. Basophils were rarely seen and hence were not included in this analysis. There were no statistically significant differences between the beagles, collie dogs, and Labrador–collie crossbreds within each group of tumour-bearing dogs, so their data were grouped together. Analysis of variance revealed that all groups of tumour-bearing dogs had significantly reduced percentages ($P < 0{\cdot}05$) and absolute ($P < 0{\cdot}05$) counts of lymphocytes when compared to the normal dogs. The mean percentages were $33{\cdot}6 \pm 0{\cdot}4\%$ for the normal dogs and $28{\cdot}7 \pm 2{\cdot}2\%$, $22{\cdot}5 \pm 2{\cdot}8\%$, and $28{\cdot}2 \pm 2{\cdot}0\%$ for the regressor, progressor, and steady-state tumour dogs respectively. The mean absolute counts of lymphocytes for the normal dogs were $4{\cdot}52 \pm 0{\cdot}20 \times 10^3/\mu l$ and $3{\cdot}36 \pm 0{\cdot}26\ \mu l$, $3{\cdot}24 \pm 0{\cdot}46\ \mu l$, and $3{\cdot}49 \pm 0{\cdot}33 \times 10^3/\mu l$ for the regressor, progressor, and steady-state dogs respectively. In contrast, there were no significant differences between the normal and tumour dogs for the total leucocyte, and for percentages and absolute counts of neutrophils, monocytes, and

TABLE II.—*Percentages and absolute counts (mean ± s.e.) of B, T, and null lymphocytes in the peripheral blood of the dogs with CTVS at different stages of growth*

| Tumour status (No.) | $SIg^+$ cells | | T Cells | | Null cells | |
|---|---|---|---|---|---|---|
| | (%) | ($\times 10^2/\mu l$) | (%) | ($\times 10^3/\mu l$) | (%) | ($\times 10^2/\mu l$) |
| Normal (12) | 17·6 ± 1·0 | 7·15 ± 0·67 | 74·7 ± 0·7 | 3·15 ± 0·15 | 7·6 ± 0·9 | 3·07 ± 0·38 |
| Regressor (23) | 17·6 ± 1·4 | 6·19 ± 0·71 | 73·1 ± 2·2 | 2·83 ± 0·43 | 9·0 ± 1·9 | 3·17 ± 0·77 |
| Progressor (11) | 14·8 ± 2·5 | 4·75 ± 0·96 | 74·4 ± 3·1 | 2·35 ± 0·78 | 11·3 ± 1·7 | 4·34 ± 0·72 |
| Steady state (10) | 17·3 ± 3·8 | 4·43 ± 1·07 | 77·0 ± 2·8 | 2·88 ± 0·34 | 9·6 ± 3·1 | 2·66 ± 0·87 |

eosinophils. Although the progressor and steady-state dogs had higher percentages and absolute monocyte counts than the normal dogs, the variation within each group was quite large and differences were not statistically significant.

*Peripheral-blood lymphocyte subpopulations*

The specificity of the immunofluorescence reagents was determined by the method described by Holden *et al.* (1976) and Wood & Neff (1978). Lymphocytes, incubated at 37°C overnight in Ig-free medium, washed × 6, incubated with heat-inactivated IgG for 1 h at 4°C, and incubated at 4°C for 45 min with FITC-conjugated rabbit anti-dog IgM serum, did not appreciably alter the percentage of $SIg^+$ cells reported below, indicating that the method used does not detect monocytes with cytophilically acquired SIgs. The rabbit anti-dog thymocyte serum has been shown in a previous study (Chandler & Yang, 1981) to be T-cell-specific and, through the use of FITC-conjugated goat anti-rabbit Ig (Alexander & Sanders, 1977), it was felt that the T-lymphocyte population was properly defined.

As shown in Table II, no significant differences in the percentages and absolute counts of B, T, and null lymphocytes were demonstrable, though the absolute counts of B cells for the progressor and steady-state dogs ($4{\cdot}75 \pm 0{\cdot}96$ and $4{\cdot}43 \pm 1{\cdot}07 \times 10^2/\mu l$ respectively) were lower than for normal dogs ($7{\cdot}15 \pm 0{\cdot}67 \times 10^2/\mu l$). The T- and B-cell absolute counts in general were lower for the tumour-bearing dogs, owing to the overall reduction of lymphocytes in these dogs (Table I).

*Draining lymph-node lymphocyte subpopulation*

The percentages of $SIg^+$, T-cell-antigen-positive, and null cells of 5 normal (2 M and 1 F collies and 1 M and 1 F beagle) and the 3 groups of tumour dogs are shown in Table III. The percentage of B cells from the draining lymph nodes of dogs with progressively growing tumours ($8{\cdot}2 \pm 2{\cdot}3\%$) was significantly lower ($P < 0{\cdot}025$) than in the normal, regressor, and steady-state tumour dogs ($16{\cdot}1 \pm 3{\cdot}1\%$, $19{\cdot}1 \pm 1{\cdot}7\%$, and $15{\cdot}8 \pm 2{\cdot}6\%$ respectively). In contrast, the percentages of T cells did not significantly differ among the groups of dogs studied. The null-cell count in the regressor dogs was lowest ($6{\cdot}9 \pm 1{\cdot}3\%$) among the groups of dogs examined, though not significantly so. Absolute numbers of the lymphocyte subpopulations in the lymph node were determined.

TABLE III.—*Percentages of B, T, and null lymphocytes (mean ± s.e.) in the draining lymph nodes of dogs with CTVS at different stages of growth*

| Tumour status (No. studied) | Cells positive for $SIg^+$ | T antigen | Null |
|---|---|---|---|
| Normal (5) | 16·1 ± 3·1* | 68·2 ± 3·7 | 13·8 ± 5·8 |
| Regressor (12) | 19·1 ± 1·7* | 74·2 ± 2·1 | 6·9 ± 1·3 |
| Progressor (5) | 8·2 ± 2·3 | 78·0 ± 1·9 | 13·8 ± 2·0 |
| Steady state (5) | 15·8 ± 2·6* | 75·1 ± 1·6 | 9·2 ± 3·4 |

* $P < 0{\cdot}025$ for progressors *vs* rest.

*Differential cell count of the tumour cell suspension*

As shown in Table IV, differential cell counts of the tumour cell suspension before density-gradient separation showed that the regressor dogs had significantly

TABLE IV.—*Differential cell counts of the cell suspensions of CTVS at different stages of growth*

| Tumour status (No.) | Tumour cell | Neutrophil | Lymphocyte | Monocyte | Eosinophil | Other |
|---|---|---|---|---|---|---|
| Regressor (23) | 72·3 ± 3·5* | 1·4 ± 0·3 | 17·3 ± 2·3* | 1·4 ± 0·3 | 0·5 ± 0·2 | 7·0 ± 2·8 |
| Progressor (11) | 91·5 ± 1·1 | 0·8 ± 0·4 | 3·4 ± 0·5 | 0·4 ± 0·1 | 0·2 ± 0·1 | 3·6 ± 0·7 |
| Steady state (7) | 91·2 ± 1·4 | 1·3 ± 0·5 | 3·3 ± 0·6 | 0·6 ± 0·3 | 0 | 3·6 ± 1·6 |

* Significantly different ($P < 0{\cdot}005$) from the other two.

TABLE V.—*Percentages of T, B, and null lymphocytes infiltrating into the CTVS at different stages of growth*

| Tumour status (No.) | $SIg^+$ | | T-antigen positive | | Null | |
|---|---|---|---|---|---|---|
| Regressor (21) | 26·1 ± 1·9 | } $P < 0·025$ | 61·2 ± 2·6 | } $P < 0·05$ | 13·5 ± 2·9 | } $P < 0·025$ |
| Progressor (10) | 37·3 ± 7·4 | | 34·0 ± 5·1 | | 26·0 ± 6·9 | |
| Steady state (9) | 30·9 ± 5·8 | | 50·8 ± 5·7 | $P < 0·005$ | 23·9 ± 8·6 | |

fewer tumour cells (72·3 ± 3·5%, $P < 0·005$) than the progressor and steady-state tumour dogs (91·5 ± 1·1% and 91·2 ± 1·4%). The regressor dogs, in contrast, had significantly more lymphocytes (17·3 ± 2·3%, $P < 0·005$) than the progressor and steady-state dogs (3·4 ± 0·5% and 3·3 ± 0·6%). The cells classified as "other" were morphologically like tumour cells, but did not have prominent nucleoli, pathognomonic of CTVS. Identification of this cell type is currently under investigation, and its significance is unclear. In contrast to other tumour systems, only 0·4–1·4% of the cells infiltrating into this tumour were monocytes.

*Subpopulations of lymphocytes infiltrating the tumour*

As shown in Table V, the percentage of B cells within the tumours of the progressor dogs (37·3 ± 7·4%) was significantly higher than in regressor dogs (26·1 ± 1·9%, $P < 0·025$). The B-cell count of steady-state dogs (30·9 ± 5·8%) lay between those of the progressors and regressors and was not significantly different from either.

The T-cell count of regressor dogs (61·2 ± 2·6%) was significantly higher than for progressor dogs (34·0 ± 5·1%, $P < 0·005$). In steady-state dogs the percentage of infiltrating T cells was 50·8 ± 5·7%, which was significantly higher than the progressors ($P < 0·005$) and significantly less than the regressors ($P < 0·005$). The null-cell count of progressor dogs (26·0 ± 6·9%) was significantly higher than that of regressor dogs (13·5 ± 2·9%, $P < 0·025$). The null-cell count of steady-state dogs was 23·9 ± 8·6%, which was not significantly different from those of either the regressor or the progressor dogs. The absolute number of infiltrating lymphocytes was not determined. However, ratios of the lymphocyte populations infiltrating regressive and progressive tumours may reflect the types of immune responses induced. For example, there were 1·8 times as many T cells within regressing tumours as in progressing ones, and 1·4 times as many B cells within progressor as in regressor tumours. Progressor tumours also had 1·9 times more null-cell infiltration than regressive ones. The percentages and ratios may indicate that regressors had more tumour-infiltrating T cells than progressors, while progressors had more B and null cells infiltrating than regressors. Steady-state dogs had intermediate values for all 3 lymphocyte populations.

### DISCUSSION

In the present study we found a parallel increase of LM cells infiltrating regressing tumours, which may be responsible, in part or in whole, for the reduced tumour size. Most infiltrating LM cells were lymphocytes. Monocyte-macrophages infiltrating into the tumour were identified by latex-bead phagocytosis and cell smears from primary cell suspensions, and the percentage of cells was found to be extremely small (Table IV). Staining of the glass dishes used to mince the tumours did not reveal adherent cells, which could have altered the percentage of macrophages, as reported. Thus most host cells in these tumours really were lymphocytes. It is felt that monocyte-macrophages do not play a major role in the immune response to this tumour, as reported by others (Korn *et al.*, 1978; Svennevig *et al.*, 1979).

Further analyses of the subpopulations of the infiltrating lymphocytes showed that the regressing tumours had higher percentages of T cells than progressive tumours, and that B cells, and possibly null cells, were more prevalent in progressive tumours. The functional role of individual lymphocyte subpopulations and their combinations in progressive tumour growth and spontaneous regression need to be established by functional studies of these lymphocytes. However, it would appear that T cells are the ones which contribute most significantly to the rejection of the tumour. It has been demonstrated that T cells are responsible for allograft rejection (Emerson, 1978) and regression of the Moloney sarcoma (Russell *et al.*, 1976).

In contrast, the prevalence of B cells in progressive tumours may underscore their role in tumour growth. For example, Wood & Neff (1978) suggested that there was an altered B-cell pattern in the presence of malignant neoplasms. This altered flow of B cells may also have been present in the dogs which had just received the tumour transplant and those which did not undergo rejection. Indeed, we have observed that the draining lymph nodes of dogs with progressively growing tumours had significantly lower percentages of B cells. Whether this tumour in its early stages of growth is chemo-attractive to B cells remains to be determined.

The significance of the null-cell population infiltrating the tumour is not known. Their identification was based on the absence of markers under study, and their number was lowest of all the cell types. However, it is of interest to note that the null-cell population fluctuated, and did not appear to parallel that of the B and T cells in the peripheral blood. All the CTVS-bearing dogs experienced a moderate lymphopenia, in which total T and B cells remained at a relatively constant level. The null-cell fluctuation may thus represent a reserve role in replenishing certain lymphocyte subpopulations.

Although absolute numbers of lymphocyte populations have not been determined, the ratios of the lymphocyte populations in the tumour and draining lymph nodes do reflect the presence of different immune responses induced by tumours at different states of growth.

These findings may suggest that B cells are preferentially attracted to progressively growing tumours and/or are stimulatory to tumours. Since there were more T cells in regressors and B cells in progressors, the presence of more null cells in the progressors suggests also that the differentiation pathway of null cells may hold the key to the fate of the tumour. However, changes in the populations of lymphocytes in the peripheral lymphoid tissues do not necessarily define the response of dogs to this tumour.

Although the present findings of changes in lymphocyte subpopulations may or may not be tumour-specific, our functional studies (*e.g.* leucocyte adherence inhibition (LAI) test (Harding & Yang, 1981)) with tumour-associated antigen (Palker & Yang, 1981) and normal tissue alloantigens, suggest that most of the changes are tumour-specific. Evaluation of the functional capacity of lymphocytes from dogs with progressive and regressive tumours is described in a subsequent paper.

This investigation was supported by Grant CA23469 awarded by the National Cancer Institute, DHHS, and is submitted as Scientific Contribution No. 835, Storrs Agricultural Experiment Station, University of Connecticut, Storrs, Connecticut 06268. We thank Ms Patricia Timmins for the preparation of the manuscript.

### REFERENCES

Alexander, E. L. & Sanders, S. K. (1977) $F(ab')_2$ reagents are not required if goat, rather than rabbit, antibodies are used to detect human surface immunoglobulin. *J. Immunol.*, **119**, 1084.

Blazar, B. A. & Heppner, G. H. (1978) *In situ* lymphoid cells of mouse mammary tumors. II. The characterization of lymphoid cells separated from mouse mammary tumors. *J. Immunol.*, **120**, 1881.

Chandler, J. P. & Yang, T. J. (1981) Identification of canine lymphocyte populations by immunofluorescence surface membrane analyses. *Int. Arch. Allergy Immunol.*, **65**, 62.

Cohen, D. (1973) The biological behavior of the transmissible venereal tumour in immunosuppressed dogs. *Eur. J. Immunol.*, **9**, 253.

COHEN, D. (1980) *In vitro* cell-mediated cytotoxicity and antibody-dependent cellular cytotoxicity to the transmissible venereal tumor of the dog. *J. Natl Cancer Inst.*, **64**, 317.

DOMAGALA, W., EMERSON, E. E. & KOSS, L. G. (1978) Distribution of T-lymphocytes and B-lymphocytes in peripheral blood and effusions of patients with cancer. *J. Natl Cancer Inst.*, **61**, 295.

EDELSON, R. L., HERRING, D. J., DELLON, A. L., FRANK, M., EDELSON, P. K. & GREEN, I. (1975) Differentiation between B cells, T cells and histiocytes in melanocytic lesions: Primary and metastatic melanoma and halo and giant pigmented nevi. *Clin. Immunol. Immunopathol.*, **4**, 557.

EMERSON, E. E. (1978) Migratory behavior of lymphocytes with specific reactivity to alloantigens. II. Selective recruitment to lymphoid cell allografts and their draining lymph node. *J. Exp. Med.*, **147**, 13.

GARNES, S. & LALA, P. K. (1978) Surface markers of small lymphocytes appearing in the mouse Ehrlich ascites tumors, host spleen and blood. *Immunology*, **34**, 487.

HARDING, M. W. & YANG, T. J. (1981) Canine transmissible venereal sarcoma: leukocyte adherence inhibition (LAI) reactivity of various lymphoid tissues of dogs with tumors at different stages of growth. *Int. J. Cancer*, **27**, 349.

HOLDEN, H. T., HASKELL, J. S., KIRCHNER, H. & HERBERMAN, R. B. (1976) Two functionally distinct anti-tumor effector cells isolated from primary murine sarcoma virus-induced tumors. *J. Immunol.*, **117**, 440.

KLEIN, E., BECKER, S., SVEDMYR, E., JONDEL, M. & VANKY, F. (1976) Tumor infiltrating lymphocytes. *Ann. N.Y. Acad. Sci.*, **276**, 207.

KLOBUSICKA, M., KALAFUT, F. & NOVOTNA, L. (1978) Studies on T and B lymphocytes in rat bearing methylcholanthrene-induced tumors. *Neoplasma*, **25**, 667.

KONORZA, G., SESTERHENN, K., KRUEGAR, G. R. F. & ABLASHI, D. V. (1979) Distribution of T- and B-cells and of immunoglobin-producing cells in tumor tissue of patients with nasopharyngeal carcinoma. *J. Cancer Res. Clin. Oncol.*, **93**, 195.

KORN, J. H., HASKILL, J. S., HOLDEN, H. T., RADOV, L. A. & RITTER, F. L. (1978) *In situ* Fc receptor-bearing cells in two murine tumors. I. Isolation and identification. *J. Natl Cancer Inst.*, **60**, 1287.

LEE, Y.-T.N., MARSHALL, G. J., WEINER, J. & BATEMAN, J. R. (1977) Peripheral B- and T-lymphocyte counts in patients with sarcoma and breast carcinoma. *Cancer*, **40**, 67.

MUSCOPLAT, C. C., SCHOSTER, J. V., OSBORNE, C. A. & JOHNSON, D. W. (1977) Density gradient separation of lymphocytes, eosinophils, and microfilariae from blood of dogs with *Dirofilaria immitis*. *Am. J. Vet. Res.*, **38**, 2095.

PALKER, T. J. & YANG, T. J. (1981) Identification and physicochemical characterization of a tumor-associated antigen from canine transmissible venereal sarcoma. *J. Natl Cancer Inst.*, **66**, 779.

RUSSELL, S. W., GILLESPIE, G. Y. & HANSEN, G. B. (1976) Inflammatory cells in solid murine neoplasms. II. Cell types found throughout the course of Moloney sarcoma regression or progression. *Int. J. Cancer*, **18**, 331.

SANTER, V., MASTROMARINO, J. H. & LALA, P. K. (1980) Characterization of the lymphocyte subsets in spontaneous mouse mammary tumors and host lymphoid organs. *Int. J. Cancer*, **25**, 159.

SILVERMAN, N. A., ALEXANDER, J. C., POTVIN, C. & CHRETIEN, P. B. (1976) *In vitro* lymphocyte reactivity and T-cell levels in patients with melanoma: Correlations with clinical and pathological stage. *Surgery*, **79**, 332.

SVENNEVIG, J. L., CLOSS, D., HARBOE, M. & SVAAR, H. (1978) Characterization of lymphocytes isolated from non-lymphoid human malignant tumours. *Scand. J. Immunol.*, **7**, 487.

SVENNEVIG, J. L., LOVIK, M. & SVAAR, H. (1979) Isolation and characterization of the lymphocytes and macrophages from solid, malignant human tumours. *Int. J. Cancer*, **23**, 626.

THYNNE, G. S., MOERTEL, C. G. & SILVERS, A. (1979) Preoperative lymphocyte counts in peripheral blood in patients with colorectal neoplasma. *Dis. Colon Rectum*, **22**, 221.

VOSE, B. M., VANKY, F. & KLEIN, E. (1977*a*) Human tumour lymphocyte interaction *in vitro*. V. Comparison of the reactivity of tumour-infiltrating, blood and lymph-node lymphocytes with autologous tumour cells. *Int. J. Cancer*, **20**, 895.

VOSE, B. M., VANKY, F., ARGOR, S. & KLEIN, E. (1977*b*) Natural cytotoxicity in man: Activity of lymph node and tumour-infiltrating lymphocytes. *Eur. J. Immunol.*, **7**, 753.

WOOD, G. W. & NEFF, J. R. (1978) A re-evaluation of B-lymphocyte levels in peripheral blood from cancer patients. *J. Natl Cancer Inst.*, **61**, 715.

YANG, T. J. & JONES, J. B. (1973) Canine transmissible venereal sarcoma: Transplantation studies in neonatal and adult dogs. *J. Natl Cancer Inst.*, **51**, 1915.

YANG, T. J., ROBERTS, R. S. & JONES, J. B. (1976) Quantitative study of lymphoreticular infiltration into canine transmissible venereal sarcoma. *Virchows Archiv.* [*Cell. Pathol.*], **20**, 197.